# The Book World of Medicine and Science

**Published:** 1907-03-09

**Authors:** 


### THE BOOK WORLD OF MEDICINE AND SCIENCE.

The Nursling: The Feeding and Hygiene of Premature and Full-term Infants. By Pierre Budin. Authorised translation by Wm. J. Maloney, M.B., Ch.B., with an Introduction by Sir Alexander Simpson, M.D. 111 diagrams in colour. (London: Caxton Publishing Company, Surrey Street, Strand.

This imposing volume, with its profusion of diagrams, is an eloquent witness to the ever-increasing attention which is being devoted to the preservation of infant life. It is only natural that France, with a birth-rate phenomenally low for many years past, should take the lead in this matter. The English birth-rate, however, is also steadily falling, whilst our infantile mortality shows, unfortunately, no corresponding diminution. It is well, therefore, that both English medical men and nurses should have an opportunity of studying the lectures of the able and well-known French professor, Dr. Budin, who by his writings and practical work has done so much to prove the eminently preventable character of the present lamentable waste of infant life. The methods which have proved so successful in his hands are minutely set forth in this volume, together with a wealth of illustrative cases, many of them in a graphic, diagrammatic form which renders it easy to grasp their significance and to avoid the study of irritating and confusing masses of figures. The pity of it is that the book should be so expensive. We are glad to note that Professor Budin emphasises the fact that breast feeding, even when artificial feeding is brought to the highest attainable pitch of perfection, must ever be the best method of rearing infants, and that when the mother's supply is inadequate it should be supplemented, and not entirely replaced, by cow's milk. Some of Professor Budin's methods differ materially from those commonly adopted in this country; for instance, he gives undiluted, sterilised cow's milk from a very early age, sometimes almost from birth, for he maintains that prolonged sterilisation so far modifies the character of the milk as to render it digestible by even quite young infants. In this country the practice of diluting the milk is still almost universally adopted, whether from prejudice, based on preconceived theories, or because pure milk has been unsuccessfully tried, is hard to say. After reading Professor Budin's strong commendation of sterilised milk it is interesting to note that only a few days ago, a deputation, which included several eminent medical men, waited upon the President of the Local Government Board to protest against the proposal to legalise the action of local authorities in establishing depots for the supply of sterilised milk for infants, on the ground that such milk is far from being an ideal food in early life. Professor Budin, on the other hand, teaches that not only is sterilised milk highly digestible, but also that its use is unattended by any risk of the development of infantile scurvy. He also condemns as inadequate the mere pasteurisation of milk, a method which has many advocates in this country. But, after all, these are only details; the essentials of Professor Budin's methods are beyond criticism. What is needed to ensure their successful adoption in this country is a higher standard of knowledge and intelligence amongst all classes of mothers, and a greater sense of the national importance of the question amongst the governing classes.

